# Differential response of esophageal cancer cells to particle irradiation

**DOI:** 10.1186/s13014-019-1326-9

**Published:** 2019-07-08

**Authors:** Sarah Hartfiel, Matthias Häfner, Ramon Lopez Perez, Alexander Rühle, Thuy Trinh, Jürgen Debus, Peter E. Huber, Nils H. Nicolay

**Affiliations:** 1 Department of Radiation Oncology, Heidelberg University Hospital, Heidelberg, Germany; 2 Department of Molecular Radiation Oncology, German Cancer Research Center (DKFZ), Heidelberg, Germany; 3 Heavy Ion Therapy Center (HIT), Heidelberg University Hospital, Im Neuenheimer Feld 450, 69120 Heidelberg, Germany; 4 Department of Radiation Oncology, University Medical Center Freiburg, University of Freiburg, Robert-Koch-Straße 3, 79106 Freiburg, Germany; 5 German Cancer Consortium (DKTK) Partner Site Freiburg, German Cancer Research Center (DKFZ), Heidelberg, Germany

**Keywords:** Esophageal cancer, Proton radiation, Carbon ion radiation, Heavy ion radiotherapy

## Abstract

**Background:**

Radiation therapy is a mainstay in the treatment of esophageal cancer (EC) patients, and photon radiotherapy has proved beneficial both in the neoadjuvant and the definitive setting. However, regarding the still poor prognosis of many EC patients, particle radiation employing a higher biological effectiveness may help to further improve patient outcomes. However, the influence of clinically available particle radiation on EC cells remains largely unknown.

**Methods:**

Patient-derived esophageal adenocarcinoma and squamous cell cancer lines were treated with photon and particle irradiation using clinically available proton (^1^H), carbon (^12^C) or oxygen (^16^O) beams at the Heidelberg Ion Therapy Center. Histology-dependent clonogenic survival was calculated for increasing physical radiation doses, and resulting relative biological effectiveness (RBE) was calculated for each radiation modality. Cell cycle effects caused by photon and particle radiation were assessed, and radiation-induced apoptosis was measured in adenocarcinoma and squamous cell EC samples by activated caspase-3 and sub-G1 populations. Repair kinetics of DNA double strand breaks induced by photon and particle radiation were investigated.

**Results:**

While both adenocarcinoma EC cell lines demonstrated increasing sensitivities for ^1^H, ^12^C and ^16^O radiation, the two squamous cell carcinoma lines exhibited a more heterogeneous response to photon and particle treatment; average RBE values were calculated as 1.15 for ^1^H, 2.3 for ^12^C and 2.5 for ^16^O irradiation. After particle irradiation, squamous cell EC samples reacted with an increased and prolonged block in G2 phase of the cell cycle compared to adenocarcinoma cells. Particle radiation resulted in an incomplete repair of radiation-induced DNA double strand breaks in both adenocarcinoma and squamous cell carcinoma samples, with the levels of initial strand break induction correlating well with the individual cellular survival after photon and particle radiation. Similarly, EC samples demonstrated heterogeneous levels of radiation-induced apoptosis that also corresponded to the observed cellular survival of individual cell lines.

**Conclusions:**

Esophageal cancer cells exhibit differential responses to irradiation with photons and ^1^H, ^12^C and ^16^O particles that were independent of tumor histology. Therefore, yet unknown molecular markers beyond histology may help to establish which esophageal cancer patients benefit from the biological effects of particle treatment.

**Electronic supplementary material:**

The online version of this article (10.1186/s13014-019-1326-9) contains supplementary material, which is available to authorized users.

## Introduction

Esophageal cancer (EC) affects more than 450,000 patients globally per year and is associated with a dismal prognosis [1]. The main histological subtypes of EC are adenocarcinomas (AC) and squamous cell carcinomas (SCC) with different etiologic factors and regional incidence patterns [1]. The current standard of care encompasses local treatments for early stage EC, often preceded by neoadjuvant therapies and multimodality treatment approaches for more frequently diagnosed locally advanced EC [2]. Despite recent advances in EC management, prognosis remains poor with estimated 5-year overall survival rates ranging between 15 to 20% [3]. Conventional photon radiotherapy (RT) is a key treatment modality for EC patients and is used both in the neoadjuvant and definitive setting, often in combination with systemic treatments [4–7]. Due to mediocre local control rates resulting in dismal patient outcome and potential severe RT-associated morbidity, there is a strong demand for improvements in RT approaches [8–10].

In contrast to conventional photon radiotherapy, particle RT offers several promising physical and biological characteristics that may be of benefit for EC patients. Charged particles exhibit an inverse dose-depth profile that allows a deposition of high radiation doses in a predefined target area (Bragg peak) while sparing surrounding healthy tissues and organs at risk. Additionally, particle RT is characterized by a higher linear energy transfer (LET) and hence an increased relative biological effectiveness compared to conventional photon RT depending on the atomic mass of the used particles and the target cells [11–13]. The higher LET leads to more densely spaced and complex DNA damage, especially irreparable DNA double-strand breaks that helps to overcome hypoxia-associated relative radiation resistance often seen in larger tumors [12]. Clinical benefits of particle beam RT have been demonstrated for several tumor entities [14–17]. However, the influence and potential benefits of particle RT on EC remain largely unexplored both in vitro and in the clinical setting, and the benefits for EC patients are unknown.

The aim of this study was to systematically evaluate the in-vitro effects of clinically available charged particles on the survival, damage response and DNA repair of EC cell lines of different histologies in comparison with photon RT.

## Materials and methods

### Cell culture

The human esophageal AC cell lines OE19 and OE33 and the human esophageal SCC lines KYSE270 and KYSE410 were purchased from the European Collection of Authenticated Cell Cultures (ECAAC, Public Health England, Salisbury, UK). OE19, OE33 and KYSE410 cells were cultured in RPMI1640 medium (Biochrom, Berlin, Germany), supplemented with 5% fetal bovine serum (Biochrom) and 1% penicillin-streptomycin (Thermo Fisher Scientific, Dreieich, Germany). KYSE270 cells were grown in 1:1 Ham’s F-12 (Biochrom) and RPMI1640 medium with the addition of 2% fetal bovine serium and 1% penicillin-streptomycin. Cells were maintained in a humidified incubator at 37 °C and 5% CO_2_.

### Radiation treatments

Photon (X) irradiation was performed with a linear accelerator (XRAD 320, Precision X-Ray, North Branford, USA) using single doses ranging between 2 and 10 Gy at 320 kV and a dose rate of 1 Gy/min. Proton (^1^H), carbon (^12^C) and oxygen (^16^O) ion irradiation was performed at the Heidelberg Ion Therapy Center using the raster-scanning technique with a spread-out Bragg peak of 35 ± 5 mm. Energy levels and linear energy transfer for ^1^H, ^12^C and ^16^O irradiation were 64.1–70 MeV/u and 6 keV/μm, 122.4–136,9 MeV/u and 101 keV/ μm, 141.4–160.9 MeV/u and 154 keV/ μm, respectively.

### Clonogenic survival assays

Cells were plated in cell culture flasks and left to attach for at least 6 h before irradiation. Cell numbers were adjusted depending on radiation quality and dose (OE19: 400–8000, OE33: 400–8000, KYSE270: 500–9000, KYSE410: 300–4000). After treatment, the cells were maintained for 10 to 14 days to allow cells to form colonies. Colonies were fixed with 25% acetic acid (v/v) in methanol and stained with crystal violet solution. Colonies with more than 50 cells were then counted by light microscopy. For each cell line and condition three independent experiments were performed with three replicate samples. The surviving fraction of cells was calculated based on the following formula: (#colonies/#plated cells)_treated_/(#colonies/#plated cells)_untreated_. Replicate samples were averaged and the mean and standard deviation (SD) of all independent experiments was plotted against radiation dose. Survival curves were fitted according to the linear-quadratic model and used to calculate the relative biological effectiveness (RBE) according to the formula: (photon dose)_10% survival_/(experimental irradiation dose)_10% survival_.

### Flow Cytometry

Cells were plated in triplicates 12 h prior to irradiation with biologically equivalent doses (2 and 8 Gy for X and ^1^H, 1 and 3 Gy for ^12^C and ^16^O) according to the mean RBE value of each irradiation modality. After 2, 8, 24, 48 and 96 h the cells were harvested using trypsin/ethylenediaminetetraacetic acid, fixed with paraformaldehyde and permeabilized with ice-cold 70% ethanol. The samples were washed three times with 0.5% bovine serum albumin (BSA) in PBS and then incubated with antibodies diluted in 3% BSA/PBS for 1 h as follows: The 2 and 8 h samples were incubated with 1:20 diluted Alexa Fluor-488-coupled antibody against γH2AX (BioLegend, San Diego, USA), the 48 and 96 h samples were incubated with 1:20 diluted Alexa Fluor-647-coupled antibody against active caspase-3 (Becton Dickinson, Heidelberg, Germany) and the 24 h samples were incubated with both antibodies at the same dilutions. All samples were then stained with 1 μg/mL DAPI (Sigma Aldrich, Taufkirchen, Germany) in PBS and measured with a LSRII flow cytometer (Beckton-Dickinson), recording 10,000 events per sample. Data analysis was performed with FlowJo 7.6.5 software (LLC, Ashland, USA) as reported before [18, 19]. Cells were gated (front scatter vs. side scatter plot) to exclude debris and cell doublets/aggregates were filtered out (DAPI-A vs. DAPI-W plot). The DAPI-A histogram of single cells was used to gate subG1 cells vs. cells with normal DNA content and to further classify the latter population into different cell cycle stages using the Dean-Jett-Fox model. G1, S and G2/M cells were gated for cell cycle-specific γH2AX measurement (DAPI-A vs. DAPI-W plot). For each population, the median γH2AX intensity was calculated and the following formula was used to calculate the combined γH2AX levels in the whole cell population (corrected for cell cycle-specific DNA content): I_A_ = I_G1_ * G1 + (I_S_ * S) / 1.5 + (I_G2|M_ * G2|M) / 2, where I_A_, I_G1_, I_S_, I_G2|M_ are median γH2AX intensities of all, G1, S, G2/M cells respectively and G1, S, G2|M are the frequencies of cells in the respective cell cycle phase. The median γH2AX levels of each population was divided by the average of the controls for normalization. For apoptosis measurement both the percentage of subG1-positive and active caspase-3-positive cells were gated in the single cell population and subtracted from the average control levels. All experiments were performed with three replicate samples.

### Statistical analyses

Statistical comparison of data was performed by two-sided Student’s t-tests using SPSS Statistics 25 software (IBM, Ehningen, Germany). These tests were paired for comparisons of clonogenic survival data between different radiation modalities or between different cell lines. All described data represent mean values and SD.

## Results

### Oesophageal cancer cell lines show heterogeneous radiation responses to particle irradiation

**Fig. 1:**
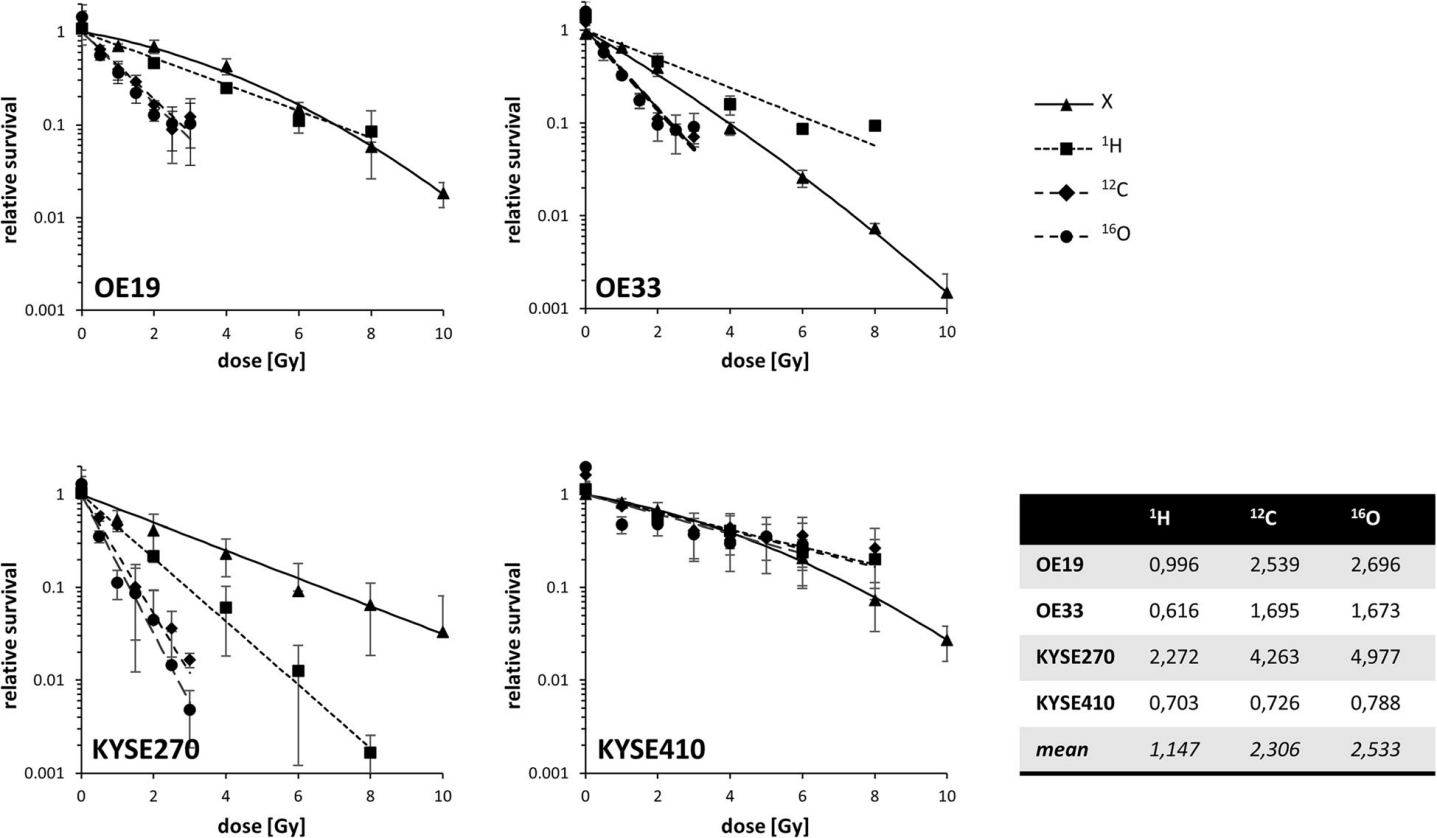
Esophageal cancer cell lines show heterogeneous survival after photon and particle irradiation. Clonogenic survival curves of OE19 and OE33 adenocarcinoma and KYSE270 and KYSE410 squamous cell carcinoma cell lines after photon (X), proton (^1^H), carbon ion (^12^C) and oxygen ion (^16^O) irradiation (mean ± SD of 3 independent experiments). The table depicts the relative biological effectiveness (RBE) for ^1^H, ^12^C and ^16^O radiation at 10% survival

Individual sensitivities of AC cell lines OE19 and OE33 and SCC cell lines KYSE270 and KYSE410 to particle radiation were assessed by clonogenic survival assays. Survival curves after exposure to conventional photon radiation were comparable for OE19, KYSE270 and KYSE410, while OE33 were markedly more sensitive, as demonstrated by a significantly stronger dose-dependent decrease in clonogenic survival (*p* = 0.001 for OE33 vs. OE19, *p* = 0.54 for OE33 vs. KYSE270, *p* < 0.001 for OE33 vs. KYSE410, two-sided paired Student’s t-test, (Additional file 1: Figure S1). For the AC cell lines OE19 and OE33, clonogenic survival did not significantly differ after photon and ^1^H irradiation (*p* = 0.09 for OE19, *p* = 0.89 for OE33, Fig. 1), whereas treatment with ^12^C and ^16^O particles resulted in significantly reduced survival in both cell lines compared to photon radiation (^12^C vs. X: *p* = 0.004 for OE19, *p* = 0.002 for OE33; ^16^O vs. X: *p* = 0.003 for OE19, *p* = 0.001 for OE33). The tested SCC cell lines showed considerable heterogeneity regarding their response to photon and particle irradiation: KYSE270 presented the highest sensitivities towards all particle irradiation modalities compared to photon treatment (*p* = 0.005 for ^1^H vs. X; *p* = 0.002 for ^12^C vs. X; *p* = 0.005 for ^16^O vs. X), while there were only marginal survival differences after photon and particle irradiation in KYSE410 cells (*p* = 0.82 for ^1^H vs. X; *p* = 0.65 for ^12^C vs. X; *p* = 0.01 for ^16^O vs. X). Resulting average RBE values at 10% survival were 1.15 for ^1^H vs. X, 2.3 for ^12^C vs. X and 2.5 for ^16^O vs. X (Fig. 1).

### Particle irradiation results in a prolonged block in the G2 phase of the cell cycle

**Fig. 2:**
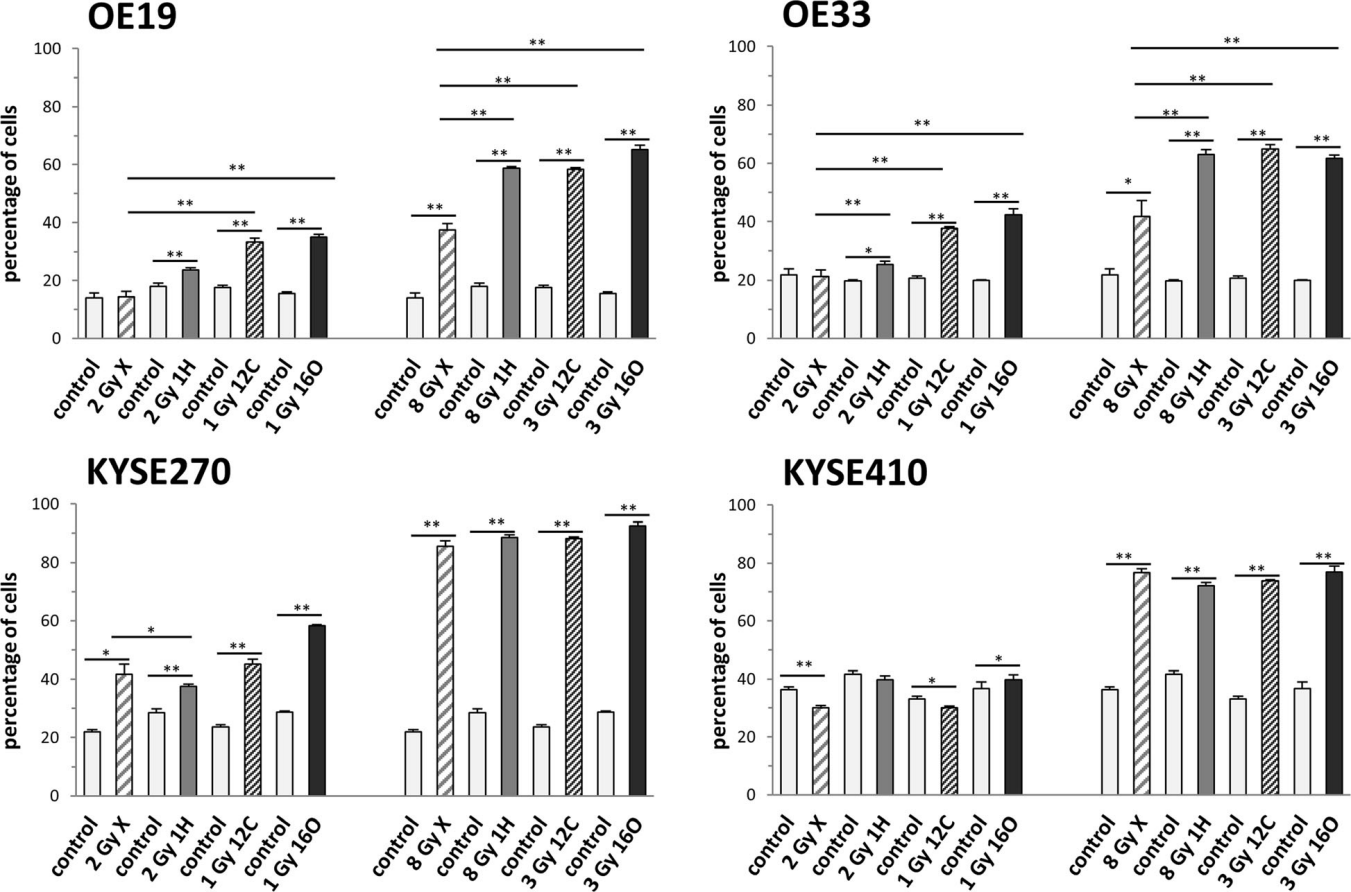
Particle irradiation results in a prolonged block in the G2 phase. Percentage of cells in G2/M phase of four EC cell lines at 24 h after irradiation with biologically isoeffective doses of photons (X), protons (^1^H) and heavy ions (^12^C, ^16^O) at a low dose level (left bars) and a high dose level (right bars) (mean and SD of *n* = 3 replicate samples). For clarity the low dose and high dose data is shown separately; the control groups are identical in both parts because for each radiation modality both dose levels were applied in the same experiment with one common control group per time point. *p < 0.05, **p < 0.01 (two-sided Student’s t-test between the indicated groups). For test comparing different radiation modalities were the control levels were subtracted from the

Flow cytometric analyses were performed to assess differential effects of photon and particle irradiation on the cell cycle of esophageal AC and SCC samples. For comparison, the cells were irradiated with isoeffective doses of photon, ^1^H, ^12^C or ^16^O radiation at a low dose level (X: 2 Gy, ^1^H: 2 Gy, ^12^C: 1 Gy, ^16^O: 1 Gy) and a high dose level (X: 8 Gy, ^1^H: 8 Gy, ^12^C: 3 Gy, ^16^O: 3 Gy). Unirradiated controls were included to determine the cell cycle distribution in unperturbed cells. Exposure to all radiation modalities resulted in a strong and prolonged block of esophageal cancer cell lines in the G2 phase of the cell cycle at the high dose level. At the low dose level, a pronounced G2 arrest was only observed in the most sensitive cell line KYSE270 for all radiation modalities and in OE19 and OE33 cells after particle irradiation. In comparison to photons, ^1^H, ^12^C and ^16^O irradiation induced a significantly stronger G2 arrest within 24 h in both AC cell lines and at both dose levels (at the high dose level X: 37%, ^1^H: 59%, ^12^C: 58%, ^16^O: 65% for OE19; X: 42%, ^1^H: 63%, ^12^C: 65%, ^16^O: 62% for OE33) (Fig. 2**,** Additional file 2: Figure S2). This pronounced G2 phase block slowly decreased, but persisted up to 96 h after particle irradiation, while AC cells exposed to photon irradiation returned to baseline levels within 96 h after treatment (Additional file 3: Figure S3, Additional file 4: Figure S4). In the SCC cell lines, a comparable to even stronger G2 phase block was observed at 24 h after treatment with either radiation modality at the high dose level, but there were no clear differences between photon and particle irradiation (X: 86%, ^1^H: 89%, ^12^C: 88%, ^16^O: 93% for KYSE270; X: 77%, ^1^H: 72%, ^12^C: 74%, ^16^O: 77% for KYSE410). In KYSE270 cells, the G2 block persisted largely unchanged up to 48 h after treatment irrespective of the radiation modality at both dose levels, correlating well with the generally high radiation sensitivity observed in the clonogenic survival assays for these cells. The more radioresistant KYSE410 cells demonstrated a swift reduction of cells arrested in G2 phase at 24 h after both photon and particle treatment at the low dose level, but a strong G2 arrest at the high dose level. At 96 h after treatment, both SCC cell lines demonstrated lower levels of G2 phase cells after photon (30% for KYSE270, 27% for KYSE410) than after ^1^H, ^12^C or ^16^O irradiation (^1^H: 48%, *p* = 0.092; ^12^C: 43%, *p* = 0.110; ^16^O: 42%, *p* = 0.038 for KYSE270; ^1^H: 42%, *p* = 0.008; ^12^C: 35%, *p* = 0.112; ^16^O: 34%, *p* = 0.006 for KYSE410).

### Photon and particle irradiation lead to heterogeneous increases in apoptosis induction of esophageal cancer cells

**Fig. 3:**
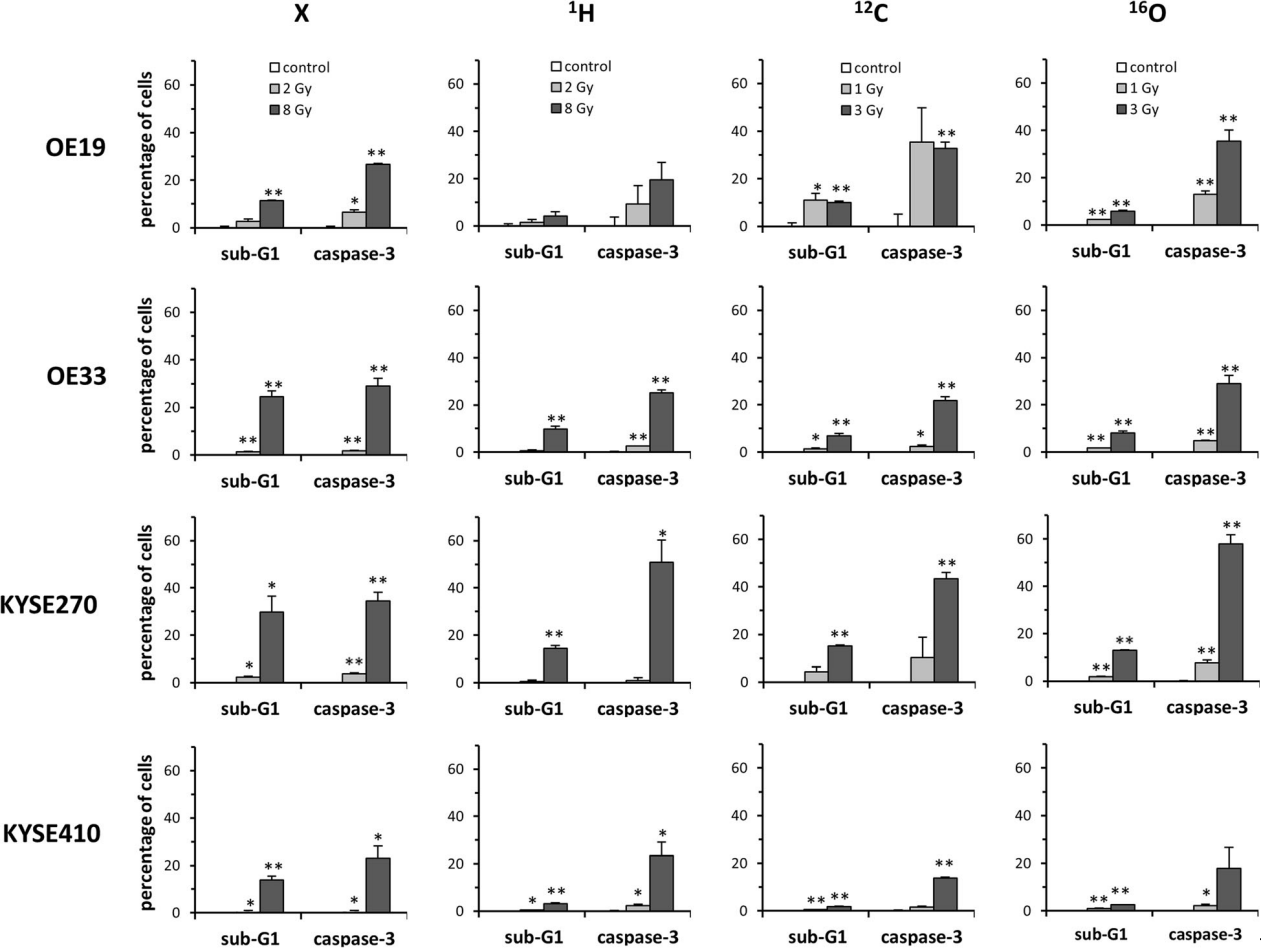
Particle irradiation leads to heterogeneous increases in apoptosis induction of esophageal cancer cells. Percentage of apoptotic EC cells accessed by the sub-G1 fraction and cellular caspase-3 activation measured at 96 h after irradiation with biologically isoeffective doses of photons (X), protons (^1^H) and heavy ions (^12^C, ^16^O) (mean and SD of *n* = 3 replicate samples). *p < 0.05, **p < 0.01 (two-sided Student’s t-test against unirradiated controls)

Cellular caspase-3 activity and sub-G1 populations were measured as indicators for early and late radiation-induced apoptosis at 48 and 96 h after irradiation with biologically isoeffective doses of photons, ^1^H, ^12^C and ^16^O particles. A time- and dose-dependent increase of apoptosis induction was detected at 48 and 96 h for all tested cell lines (Fig. 3**,** Additional file 5: Figures S5, Additional file 6: Figure S6). While lower isoeffective doses of 2 Gy photons and ^1^H and 1 Gy ^12^C and ^16^O resulted in apoptosis induction in less than 5% of tested cell lines, a time- and dose-dependent increase of apoptosis induction was detected at 48 and 96 h for the high dose level. At 96 h after irradiation, comparable rates of caspase-3-positive cells ranging between 28 and 35% were found for all tested radiation modalities; however, the average apoptotic sub-G1 population after photon radiation amounted to 20% and was significantly higher than after ^1^H (8%, *p* < 0.05), ^12^C (8%, *p* < 0.05) and ^16^O radiation (7%, *p* < 0.05). Considerable heterogeneity regarding apoptosis induction was noted for the SCC cell lines: KYSE270 demonstrated higher apoptosis levels after ^12^C (*p* < 0.05) and ^16^O (*p* < 0.01) irradiation than after photon treatment as measured by caspase-3 activation, whereas KYSE410 demonstrated lower apoptosis rates for ^12^C and ^16^O particle radiation compared to conventional photons, although statistical significance was not reached (*p* = 0,095 for ^12^C, *p* = 0,44 for ^16^O); this correlated well with the clonogenic survival data observed after photon and particle irradiation in these cell lines. The AC cell lines did not exhibit a general difference in apoptosis induction between photons and particle radiation. OE19 cells demonstrated higher levels of caspase-3 activation after particle than after photon irradiation at 48 and 96 h that were not reflected in the sub-G1 population of these cells. In contrast, the sub-G1 population of OE33 cells exhibited an increased sub-G1 population after photon irradiation compared to particle treatment after 48 and 96 h that was not mirrored in increased caspase-3 activation.

### DNA double strand breaks induced by particle radiation are incompletely repaired

**Fig. 4:**
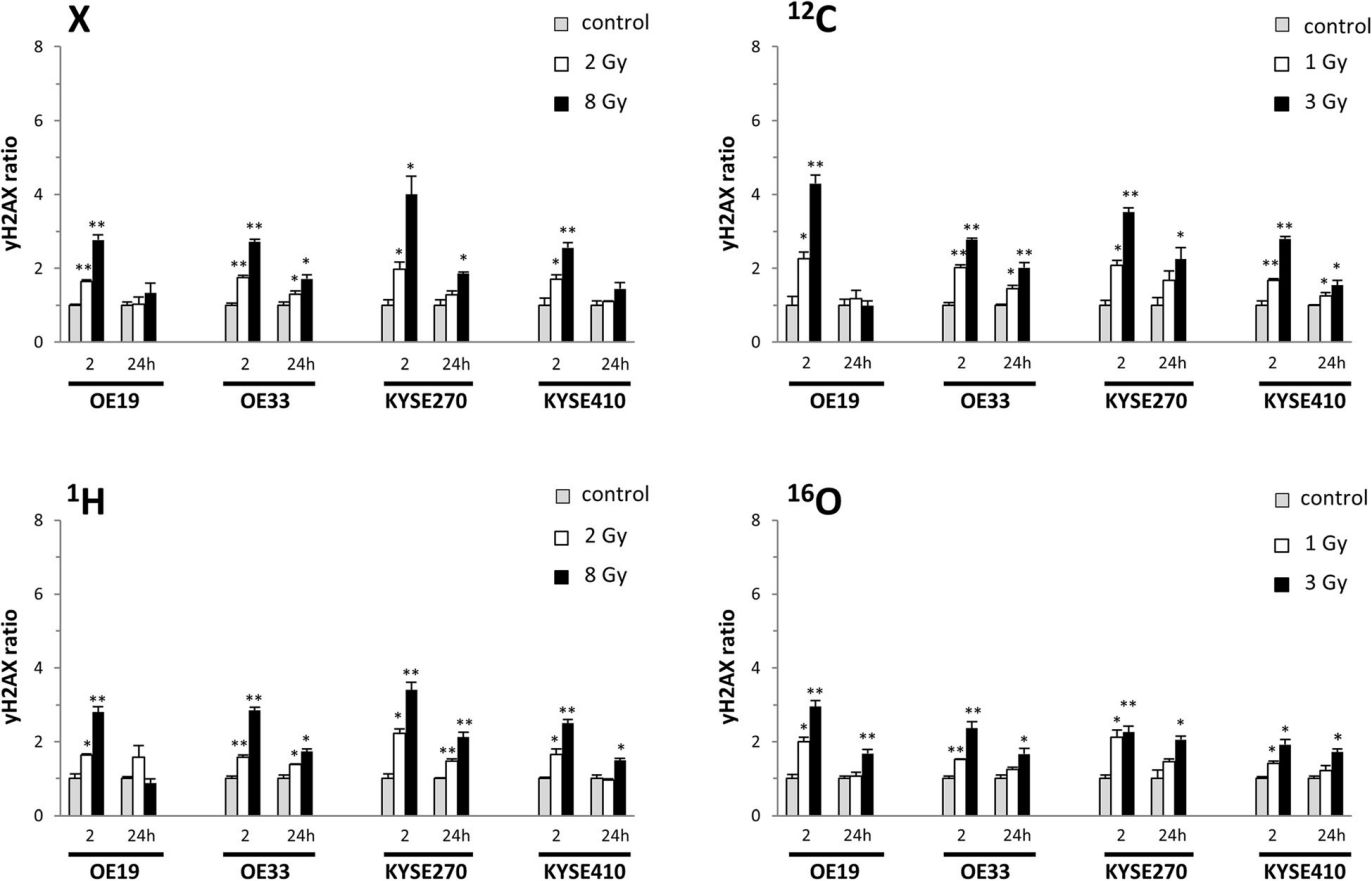
Esophageal cancer cells incompletely repair DNA double strand breaks induced by particle irradiation. Normalized γH2AX levels at 2 and 24 h after irradiation with biologically isoeffective doses of photons (X), protons (^1^H) and heavy ions (^12^C, ^16^O) (mean and SD of *n* = 3 replicate samples). Values were corrected for cell cycle-specific differences as detailed in Materials and Methods. *p < 0.05, **p < 0.01 (two-sided Student’s t-test against unirradiated controls)

Levels of the phosphorylated H2AX histone protein (γH2AX) were measured in a cell cycle- specific way by flow cytometry as a surrogate marker for radiation-induced DNA double strand breaks. Photon irradiation resulted in a swift increase of γH2AX peak levels within 2 h in all tested esophageal cancer cells (Fig. 4**,** Additional files 7, 8, 9 and 10: Figures S7-S10). While γH2AX levels returned to baseline after low photon doses of 2 Gy, indicating complete repair of double strand breaks, peak levels were found incompletely resolved after higher photon doses of 8 Gy, indicating unrepaired radiation-induced DNA damage. Similar γH2AX kinetics were observed after ^1^H irradiation with high peak levels after 2 h and incomplete resolution of H2AX phosphorylation within 24 h for all tested cell lines. For ^12^C radiation, a significant decrease of γH2AX levels between 2 and 24 h was only measured for OE19 cells, while all other cell lines exhibited no significant reduction of peak γH2AX levels, suggesting negligible repair of radiation-induced damage. Comparable lack of DNA double strand break repair was observed after ^16^O irradiation for all esophageal cell lines. Of note, KYSE410 cells continuously exhibited the lowest γH2AX levels at 2 h after irradiation, most likely indicating very efficient DSB repair already within this rather short time period, corresponding to their pronounced resistance to both photon and particle irradiation.

## Discussion

Here, we demonstrated for the first time the differential effects of photon and particle irradiation on esophageal cancer cell lines of different histologies regarding cellular survival, apoptosis induction and DNA double strand break repair.

Clinically available ion beam radiation with protons or heavier ions like ^12^C or ^16^O may make use of the particles’ physical advantages like their inverted dose-depth profile, resulting in the ability to increase treatment doses while sparing surrounding organs-at-risk, or their increased RBE values. Indeed, in our dataset, the RBE values for ^12^C and ^16^O radiation were determined to be 2.3 and 2.5, respectively. These values compare well with previous RBE calculations of ^12^C irradiation obtained from animal models that have suggested values around 2.0 compared to photon irradiation [18, 19]. No data have been published yet concerning RBE values of particle radiation using heavier ions such as ^16^O radiation in esophageal cancer.

We found significant differences in cellular sensitivities to photon and particle radiation in the two human esophageal SCC cell lines tested, while the survival data were more consistent for the 2 AC cell lines. Histology-dependent radiation responses have been widely reported for esophageal cancer not only in vitro, but even in patients undergoing clinical radiation therapy [4, 20, 21]. Esophageal SCC is known to be more radiosensitive compared to AC, resulting in a higher rate of pathological complete response (pCR) after neoadjuvant chemoradiotherapy. Albeit pCR rate is higher for esophageal SCC, both SCC and AC esophageal cancer benefit from preoperative chemoradiotherapy. However, factors beyond tumor histopathology may strongly influence radiation responses of individual esophageal cancers not only to photon but especially particle radiation, and indeed, our data suggest a strong heterogeneity of RBE values that were not histology-specific: RBE values for ^12^C and ^16^O radiation ranged between 0.7 and 4.3 and 0.8 and 5.0, respectively, with the SCC cell lines exhibiting both the most radioresistant and radiosensitive phenotypes.

Surprisingly, OE33 cells were observed to be more resistant to photon compared to proton irradiation. Additionally, KYSE410 exhibited similar clonogenic survival curves for ^1^H, ^12^C and ^16^O radiation. Resistance to proton and particle irradiation is linked to effective homologous recombination (HR) DSB repair, and previous studies have shown the important role of HR for esophageal AC and SCC such as OE33 and KYSE410 [22–25]. However, further studies are needed to elucidate the mechanisms for the increased resistance to proton and particle irradiation in these cell lines.

All tested types of particle irradiation resulted in incomplete repair of radiation-induced DNA double strand breaks, correlating with a prolonged G2 phase arrest. Cell cycle arrests are usually signs of ongoing attempts of the cell to repair life-threatening DNA lesions such as DSBs, but are not necessarily predictive for the success of DNA repair. Previous publications have demonstrated correlations of residual γH2AX signals detectable beyond 24 h after irradiation with both increased radiation sensitivity and increased DNA repair capacity [26–28]. In line with these reports, both the radiosensitive KYE270 and the strongly resistant KYSE410 cell lines were found to exhibit strong increases in G2 phase cells with elevated γH2AX levels at 24 h after treatment in our dataset. Additionally, response to particle irradiation was found to correlate with the level of apoptosis induction in all tested esophageal cancer samples, and the cell lines most sensitive to ^12^C and ^16^O irradiation exhibited the highest levels of caspase-3 induction at 96 h after treatment. Interestingly, caspase-3 activation was higher after ^12^C and ^16^O irradiation versus photons in OE19 and KYSE270 cells, despite of isoeffective dosage, while only marginal differences were observed in OE33 cells and slightly smaller levels were seen for KYSE410 cells irradiated with ^12^C and ^16^O versus X and ^1^H. These variations might indicate that other types of cell death contribute to the overall cell killing effect in a cell line- and radiation modality-dependent manner. Previous reports have outlined a correlation between the induction of apoptosis and the response of esophageal cancer cells and patients to radiation therapy [29–31]. However, it remains unclear if apoptosis levels are linked to the prognostic rates of pathological complete remission after irradiation. Considering the increased biological effectiveness of particle irradiation, especially with heavier ^12^C or ^16^O beams and the superior physical dose distribution of these particle treatments, it is conceivable that distinct patient subgroups may derive a clinical benefit from ^12^C or ^16^O-based radiation therapy. However, the identification of these patient subgroups does not seem to rely on the histology, and additional molecular markers and signatures are warranted to define the individual benefit of each patient to this novel treatment. While this analysis investigated the use of particle radiation alone on esophageal cancer cells in order to quantify the biological effects, clinical treatment algorithms rely on the combination of radiation with systemic treatments including potentially radiosensitizing anti-cancer drugs such as platinum compounds, 5-fluorouracil or paclitaxel [4, 6, 32]. Therefore, combination treatments remain to be investigated in order to establish a potential use of particle irradiation in clinically established chemo-radiotherapy protocols.

## Conclusions

Taken together, we demonstrated a differential sensitivity of esophageal cancer cells to irradiation with photons and ^1^H, ^12^C and ^16^O particles that was independent of tumor histology. Considering the vastly different responses of esophageal cancer samples to particle irradiation, yet unknown molecular markers may help to establish which esophageal cancer patients may benefit from particle treatments.

## Additional files

Additional file 1: **Figure S1.** Survival curves after exposure to photon irradiation. Linear-quadratic fits of clonogenic survival data from OE19 and OE33 adenocarcinoma and KYSE270 and KYSE410 squamous cell carcinoma cell lines after photon. (TIF 449 kb)

Additional file 2: **Figure S2.** Particle irradiation results in a prolonged block in the G2 phase. Cell cycle profiles of four EC cell lines at 24 h after irradiation with biologically isoeffective doses of photons (X), protons (^1^H) and heavy ions (^12^C, ^16^O) (mean and SD of *n* = 3 replicate samples). *p < 0.05, **p < 0.01 (two-sided Student’s t-test against unirradiated controls). (TIF 701 kb)

Additional file 3: **Figure S3.** G2 phase arrest at 48 h after particle irradiation. Cell cycle distribution of EC cell lines at 48 h after irradiation with biologically isoeffective doses of photons (X), protons (^1^H) and heavy ions (^12^C, ^16^O) (mean and SD of *n* = 3 replicate samples).*p < 0.05, **p < 0.01 (two-sided Student’s t-test against unirradiated controls). (TIF 699 kb)

Additional file 4: **Figure S4.** G2 phase arrest at 96 h after particle irradiation. Cell cycle distribution of EC cell lines at 96 h after irradiation with biologically isoeffective doses of photons (X), protons (^1^H) and heavy ions (^12^C, ^16^O) (mean and SD of *n* = 3 replicate samples). *p < 0.05, **p < 0.01 (two-sided Student’s t-test against unirradiated controls). (TIF 702 kb)

Additional file 5: **Figure S5.** Apoptosis induction in different esophageal cancer cell lines at 24 h after treatment with different radiation modalities. Percentage of apoptotic EC cells as accessed by the sub-G1 fraction and cellular caspase-3 activity at 24 h after irradiation with biologically isoeffective doses of photons (X), protons (^1^H) and heavy ions (^12^C, ^16^O) (mean and SD of *n* = 3 replicate samples). *p < 0.05, **p < 0.01 (two-sided Student’s t-test against unirradiated controls). (TIF 586 kb)

Additional file 6: **Figure S6.** Particle irradiation induces varying levels of apoptosis in different esophageal cancer cell lines at 48 h after irradiation. Percentage of apoptotic EC cells as accessed by the sub-G1 fraction and cellular caspase-3 activity at 48 h after irradiation with biologically isoeffective doses of photons (X), protons (^1^H) and heavy ions (^12^C, ^16^O) (mean and SD of *n* = 3 replicate samples). *p < 0.05, **p < 0.01 (two-sided Student’s t-test against unirradiated controls). (TIF 613 kb)

Additional file 7: **Figure S7.** Induction and repair of DNA double strand breaks in G1 phase cells after irradiation. Normalized γH2AX levels of G1 phase cells at 2, 8 and 24 h after irradiation with biologically isoeffective doses of photons (X), protons (^1^H) and heavy ions (^12^C, ^16^O) (mean and SD of *n* = 3 replicate samples). *p < 0.05, **p < 0.01 (two-sided Student’s t-test against unirradiated controls). (TIF 592 kb)

Additional file 8: **Figure S8.** Induction and repair of DNA double strand breaks in S phase cells after irradiation. Normalized γH2AX levels of S phase cells at 2, 8 and 24 h after irradiation with biologically isoeffective doses of photons (X), protons (^1^H) and heavy ions (^12^C, ^16^O) (mean and SD of *n* = 3 replicate samples). *p < 0.05, **p < 0.01 (two-sided Student’s t-test against unirradiated controls). (TIF 570 kb)

Additional file 9: **Figure S9.** Induction and repair of DNA double strand breaks in G2 phase cells after irradiation. Normalized γH2AX levels of G2 phase cells at 2, 8 and 24 h after irradiation with biologically isoeffective doses of photons (X), protons (^1^H) and heavy ions (^12^C, ^16^O) (mean and SD of *n* = 3 replicate samples). *p < 0.05, **p < 0.01 (two-sided Student’s t-test against unirradiated controls). (TIF 568 kb)

Additional file 10: **Figure S10.** Induction and repair of DNA double strand breaks in esophageal cancer cells after irradiation. γH2AX levels (not normalized) at 2 and 24 h after irradiation with biologically isoeffective doses of photons (X), protons (^1^H) and heavy ions (^12^C, ^16^O) (mean and SD of *n* = 3 replicate samples). Values were corrected for cell cycle-specific differences as detailed in Materials and Methods. *p < 0.05, **p < 0.01 (two-sided Student’s t-test against unirradiated controls). (TIF 602 kb)

## Data Availability

The datasets used and analyzed during the current study are available from the corresponding author on reasonable request.
