# Supplementary figures and images for: Differential response of esophageal cancer cells to particle irradiation
Source: Radiat Oncol. 2019 Jul 8;14:119. doi: 10.1186/s13014-019-1326-9 (PMC6615091; doi:10.1186/s13014-019-1326-9)

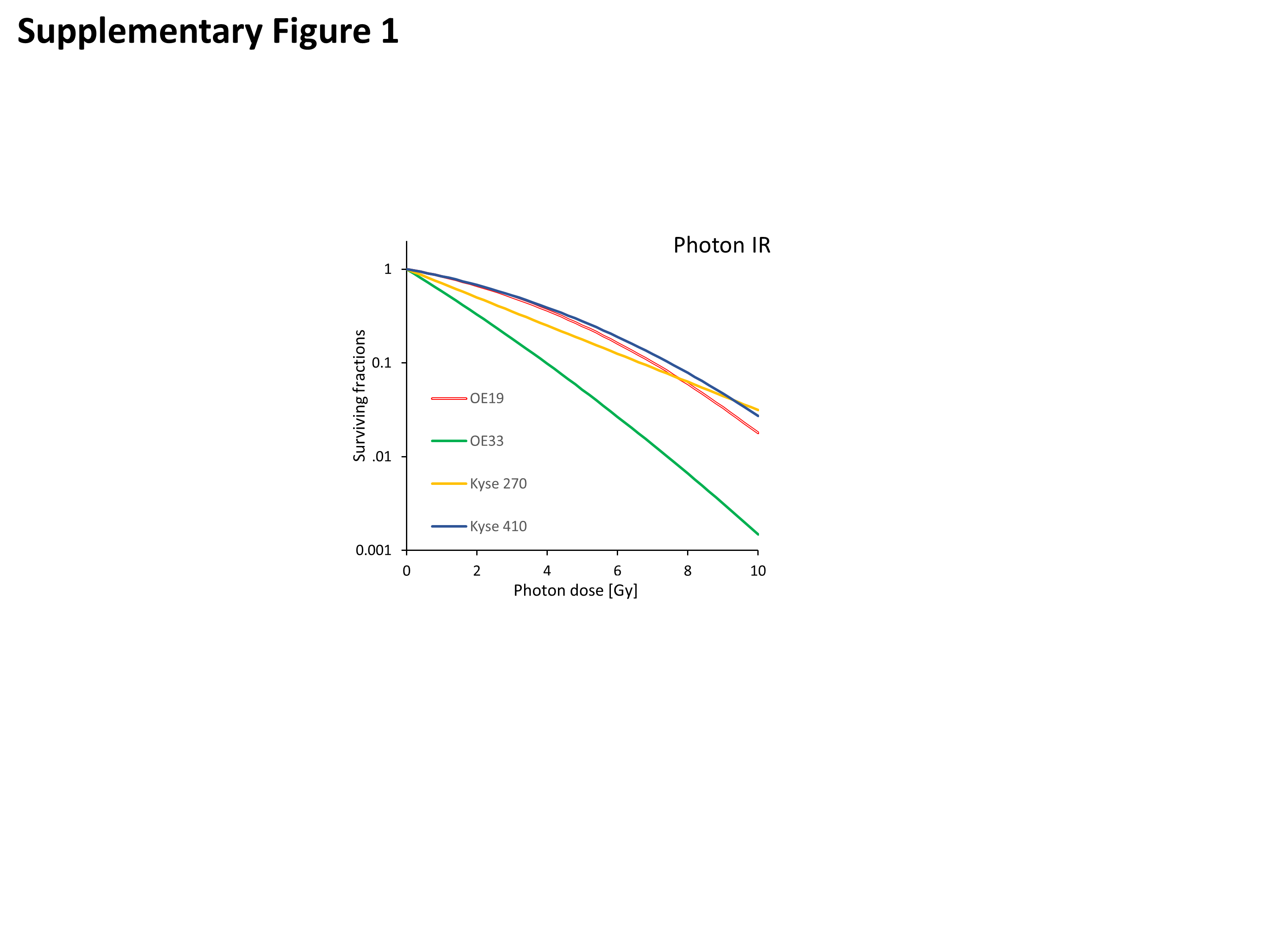

Supplement: Supplementary file 1 — Figure S1. Survival curves after exposure to photon irradiation. Linear-quadratic fits of clonogenic survival data from OE19 and OE33 adenocarcinoma and KYSE270 and KYSE410 squamous cell carcinoma cell lines after photon. (TIF 449 kb) [file 13014_2019_1326_MOESM1_ESM.tif]

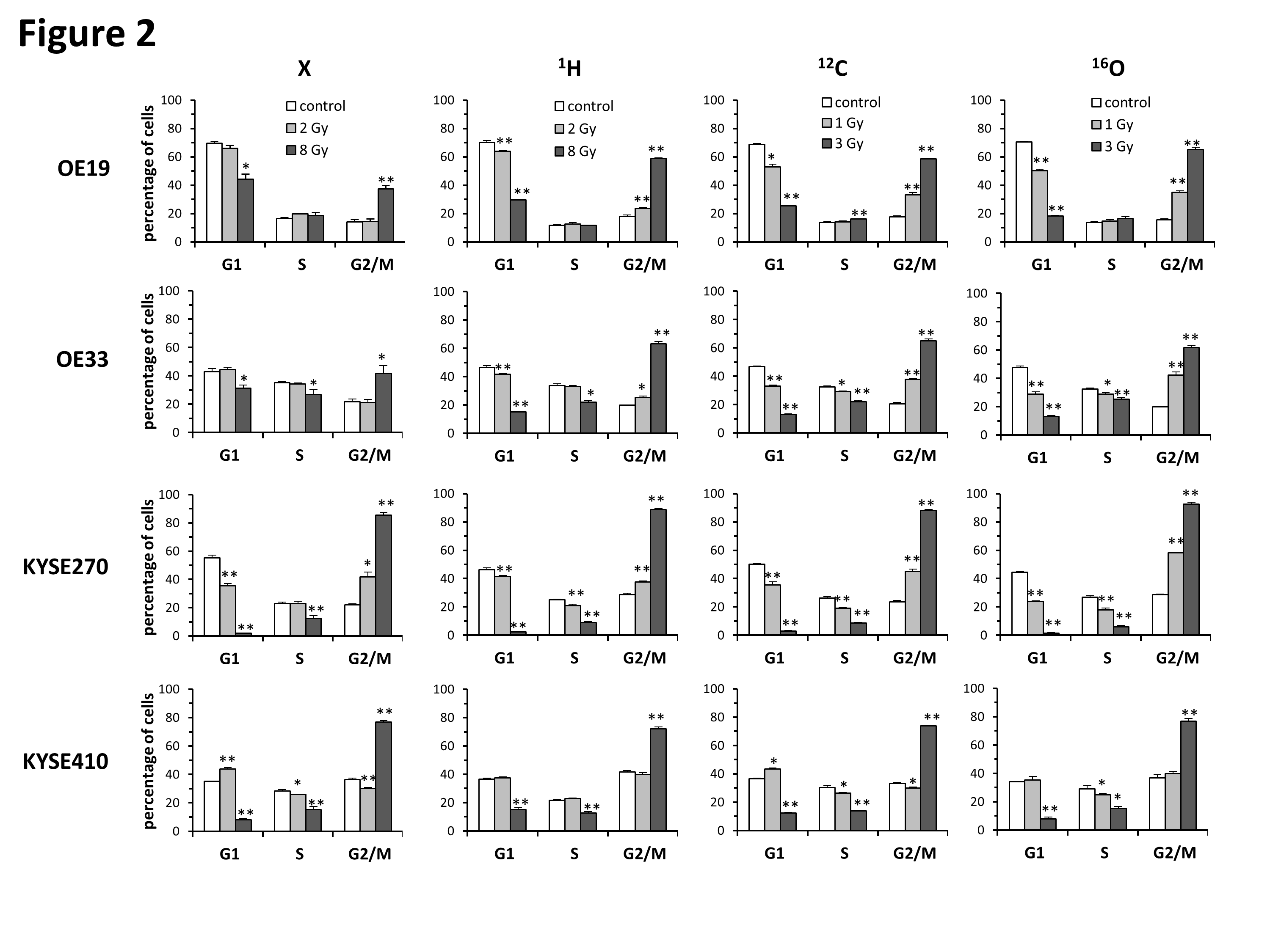

Supplement: Supplementary file 2 — Figure S2. Particle irradiation results in a prolonged block in the G2 phase. Cell cycle profiles of four EC cell lines at 24 h after irradiation with biologically isoeffective doses of photons (X), protons (1H) and heavy ions (12C, 16O) (mean and SD of n = 3 replicate samples). *p < 0.05, **p < 0.01 (two-sided Student’s t-test against unirradiated controls). (TIF 701 kb) [file 13014_2019_1326_MOESM2_ESM.tif]

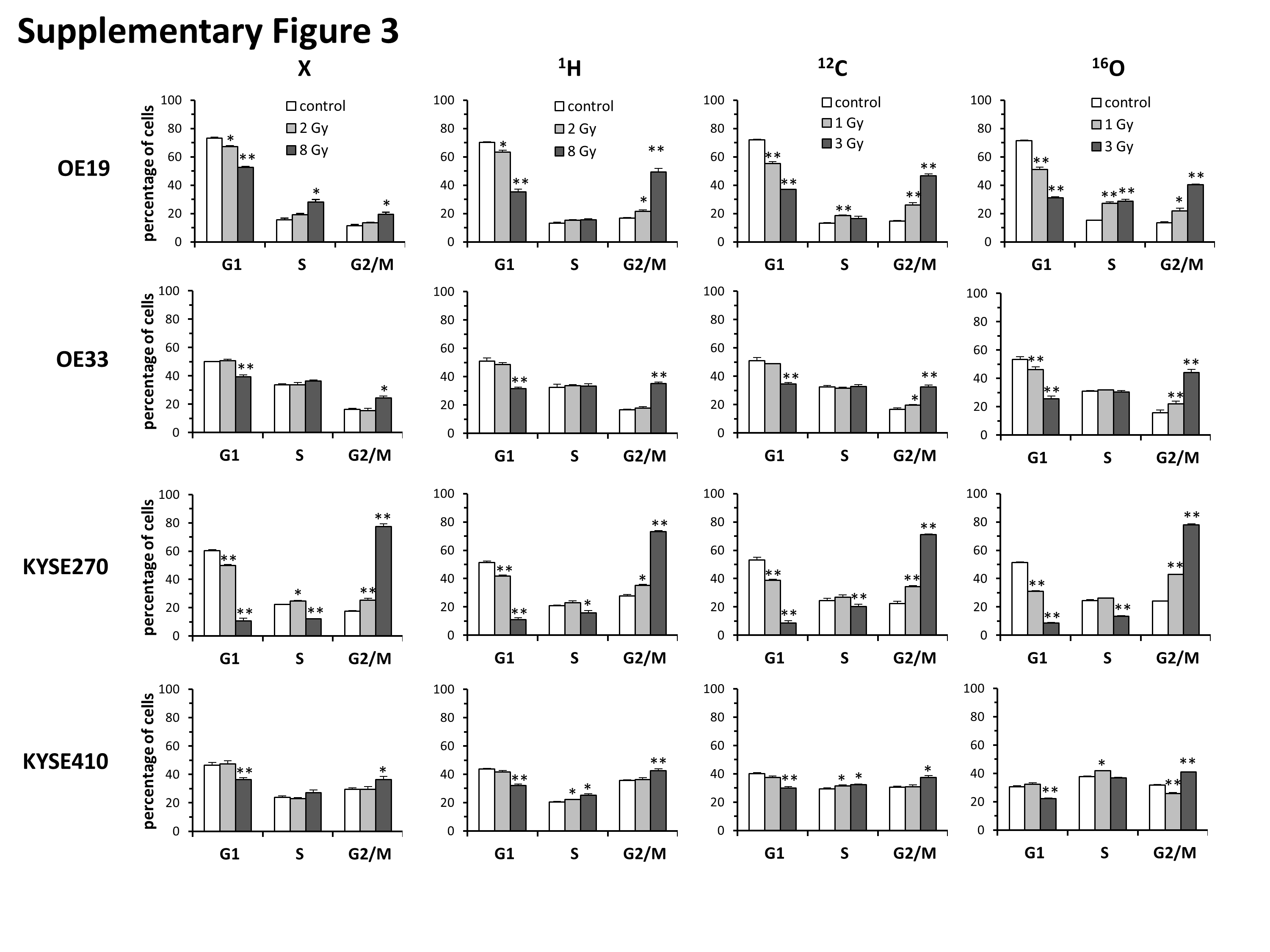

Supplement: Supplementary file 3 — Figure S3. G2 phase arrest at 48 h after particle irradiation. Cell cycle distribution of EC cell lines at 48 h after irradiation with biologically isoeffective doses of photons (X), protons (1H) and heavy ions (12C, 16O) (mean and SD of n = 3 replicate samples).*p < 0.05, **p < 0.01 (two-sided Student’s t-test against unirradiated controls). (TIF 699 kb) [file 13014_2019_1326_MOESM3_ESM.tif]

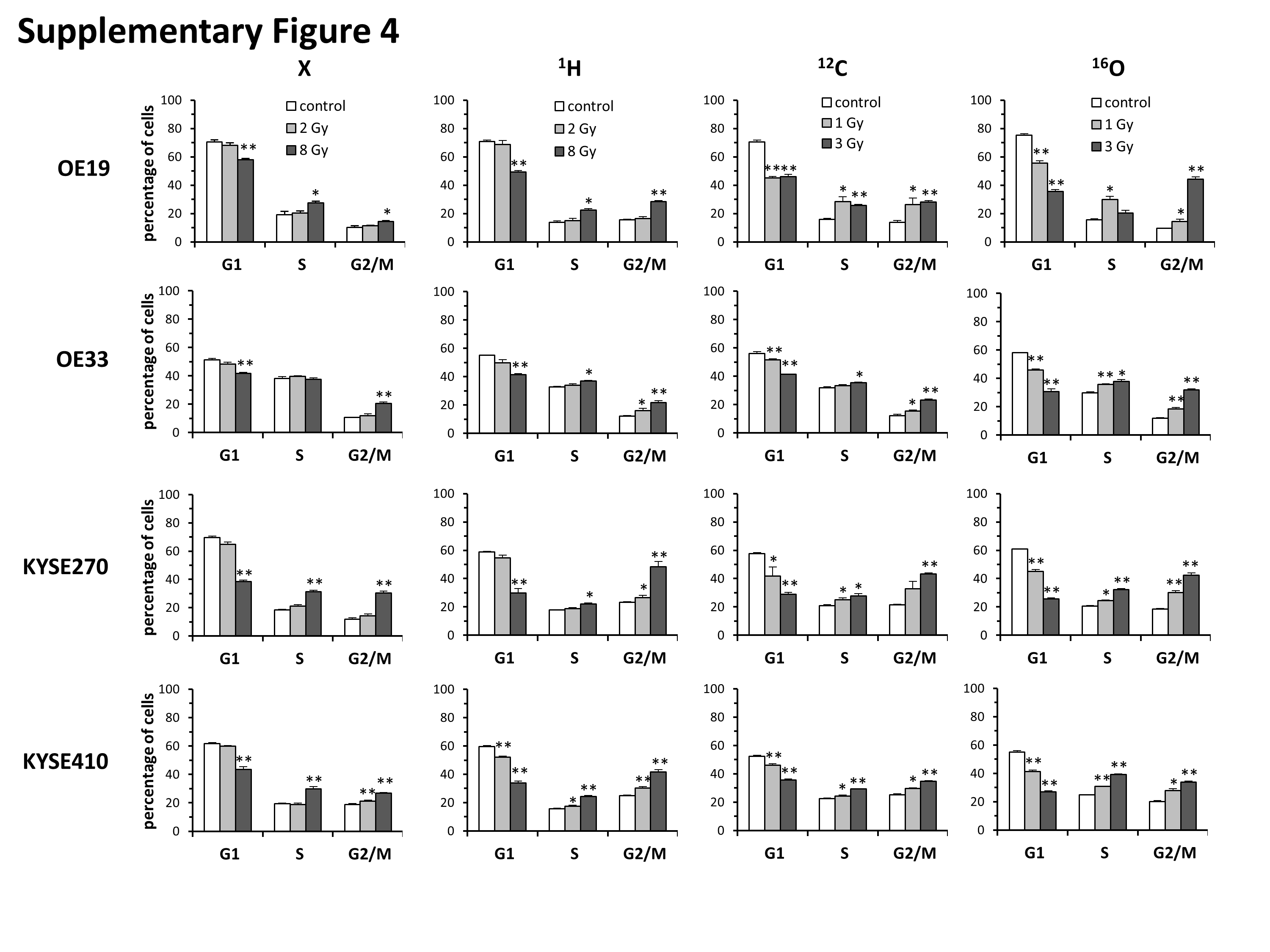

Supplement: Supplementary file 4 — Figure S4. G2 phase arrest at 96 h after particle irradiation. Cell cycle distribution of EC cell lines at 96 h after irradiation with biologically isoeffective doses of photons (X), protons (1H) and heavy ions (12C, 16O) (mean and SD of n = 3 replicate samples). *p < 0.05, **p < 0.01 (two-sided Student’s t-test against unirradiated controls). (TIF 702 kb) [file 13014_2019_1326_MOESM4_ESM.tif]

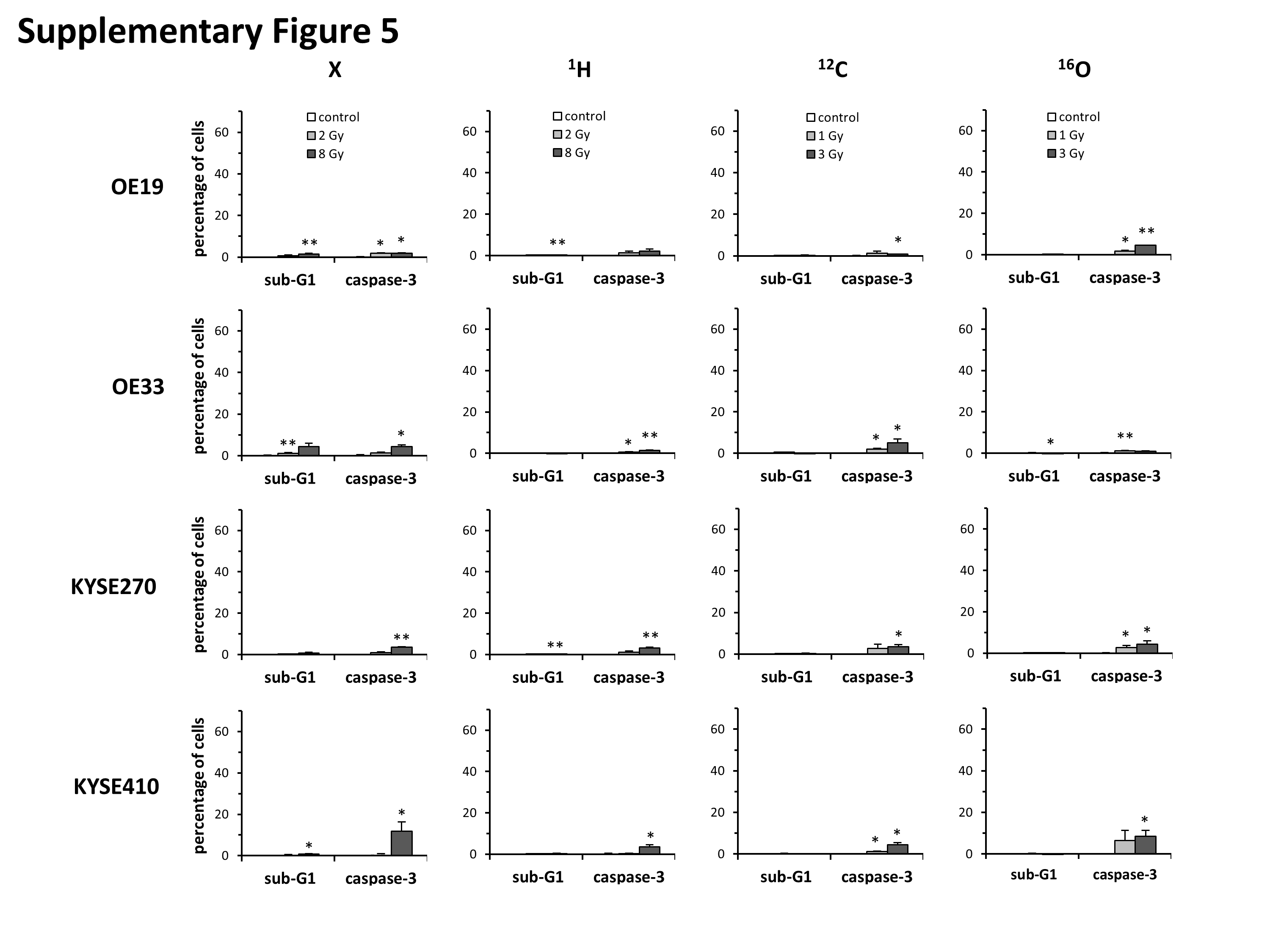

Supplement: Supplementary file 5 — Figure S5. Apoptosis induction in different esophageal cancer cell lines at 24 h after treatment with different radiation modalities. Percentage of apoptotic EC cells as accessed by the sub-G1 fraction and cellular caspase-3 activity at 24 h after irradiation with biologically isoeffective doses of photons (X), protons (1H) and heavy ions (12C, 16O) (mean and SD of n = 3 replicate samples). *p < 0.05, **p < 0.01 (two-sided Student’s t-test against unirradiated controls). (TIF 586 kb) [file 13014_2019_1326_MOESM5_ESM.tif]

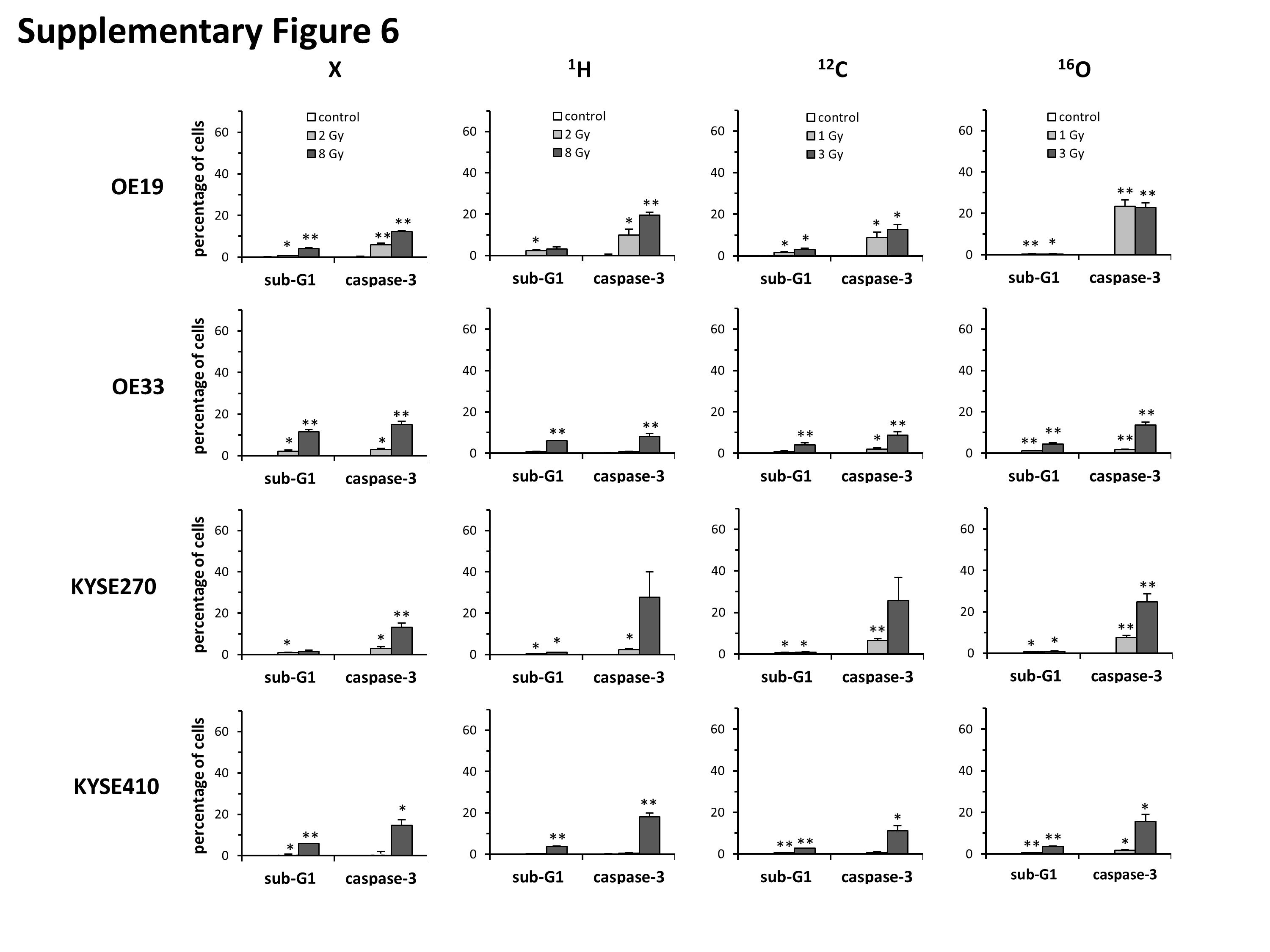

Supplement: Supplementary file 6 — Figure S6. Particle irradiation induces varying levels of apoptosis in different esophageal cancer cell lines at 48 h after irradiation. Percentage of apoptotic EC cells as accessed by the sub-G1 fraction and cellular caspase-3 activity at 48 h after irradiation with biologically isoeffective doses of photons (X), protons (1H) and heavy ions (12C, 16O) (mean and SD of n = 3 replicate samples). *p < 0.05, **p < 0.01 (two-sided Student’s t-test against unirradiated controls). (TIF 613 kb) [file 13014_2019_1326_MOESM6_ESM.tif]

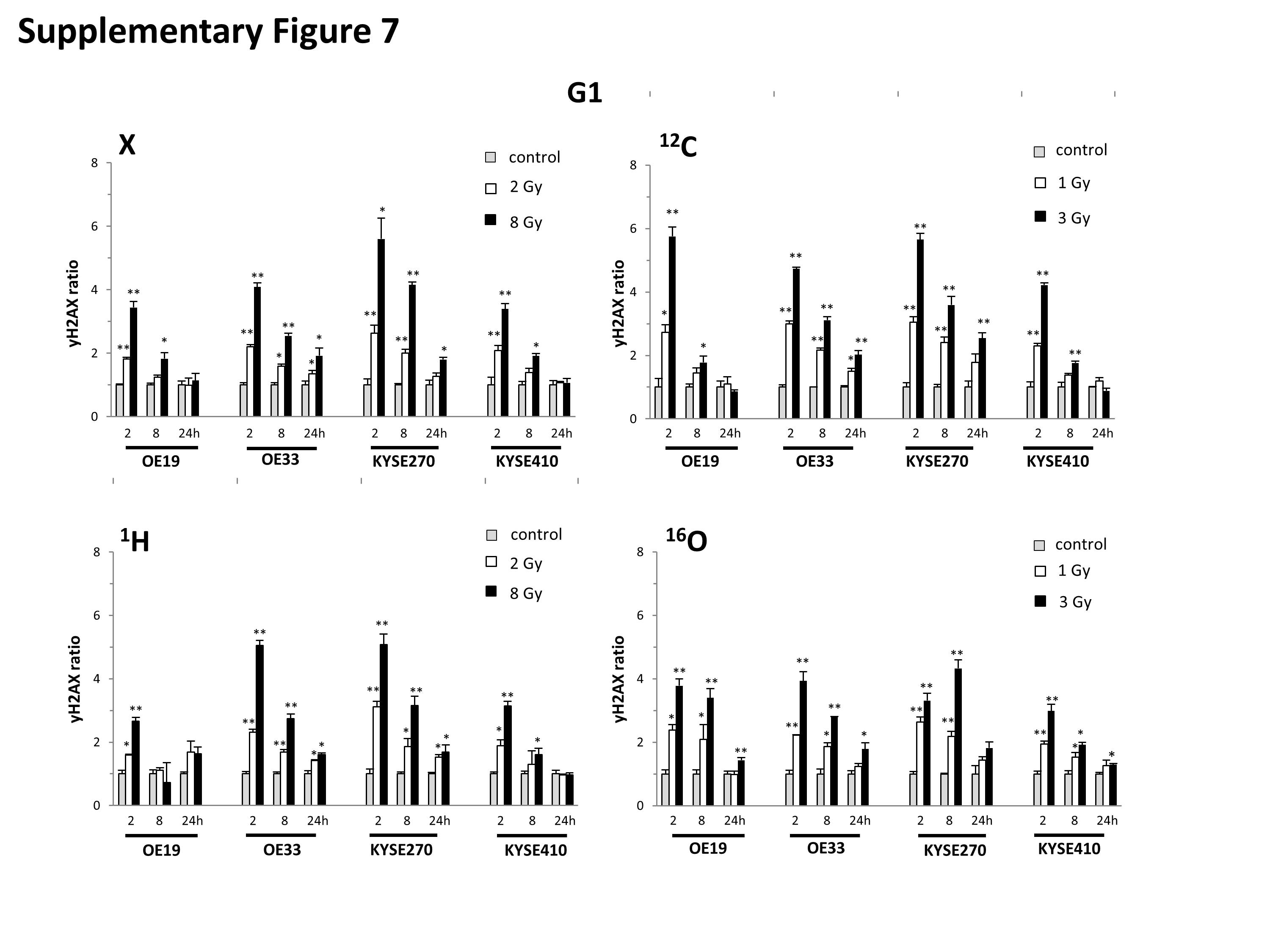

Supplement: Supplementary file 7 — Figure S7. Induction and repair of DNA double strand breaks in G1 phase cells after irradiation. Normalized γH2AX levels of G1 phase cells at 2, 8 and 24 h after irradiation with biologically isoeffective doses of photons (X), protons (1H) and heavy ions (12C, 16O) (mean and SD of n = 3 replicate samples). *p < 0.05, **p < 0.01 (two-sided Student’s t-test against unirradiated controls). (TIF 592 kb) [file 13014_2019_1326_MOESM7_ESM.tif]

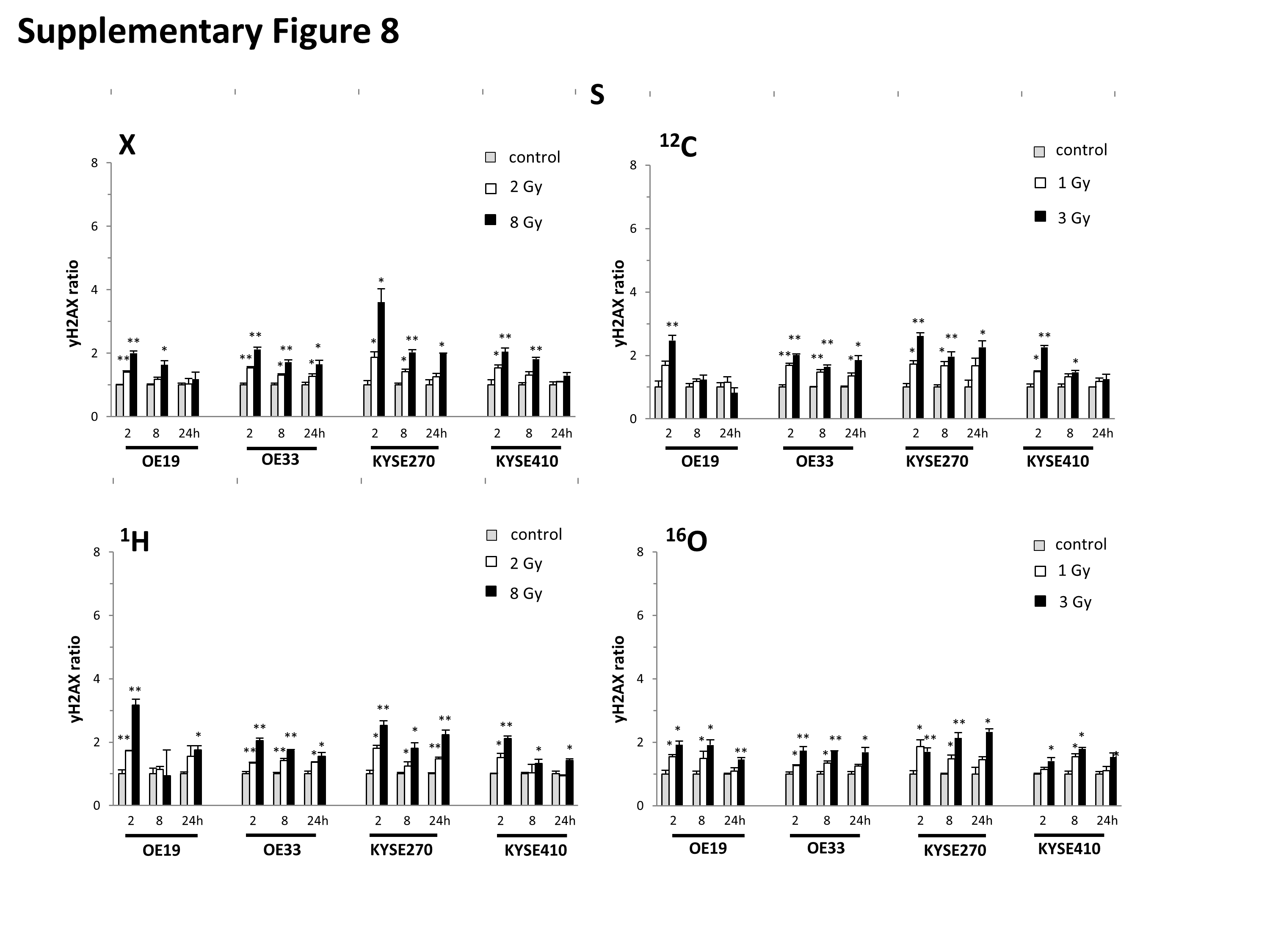

Supplement: Supplementary file 8 — Figure S8. Induction and repair of DNA double strand breaks in S phase cells after irradiation. Normalized γH2AX levels of S phase cells at 2, 8 and 24 h after irradiation with biologically isoeffective doses of photons (X), protons (1H) and heavy ions (12C, 16O) (mean and SD of n = 3 replicate samples). *p < 0.05, **p < 0.01 (two-sided Student’s t-test against unirradiated controls). (TIF 570 kb) [file 13014_2019_1326_MOESM8_ESM.tif]

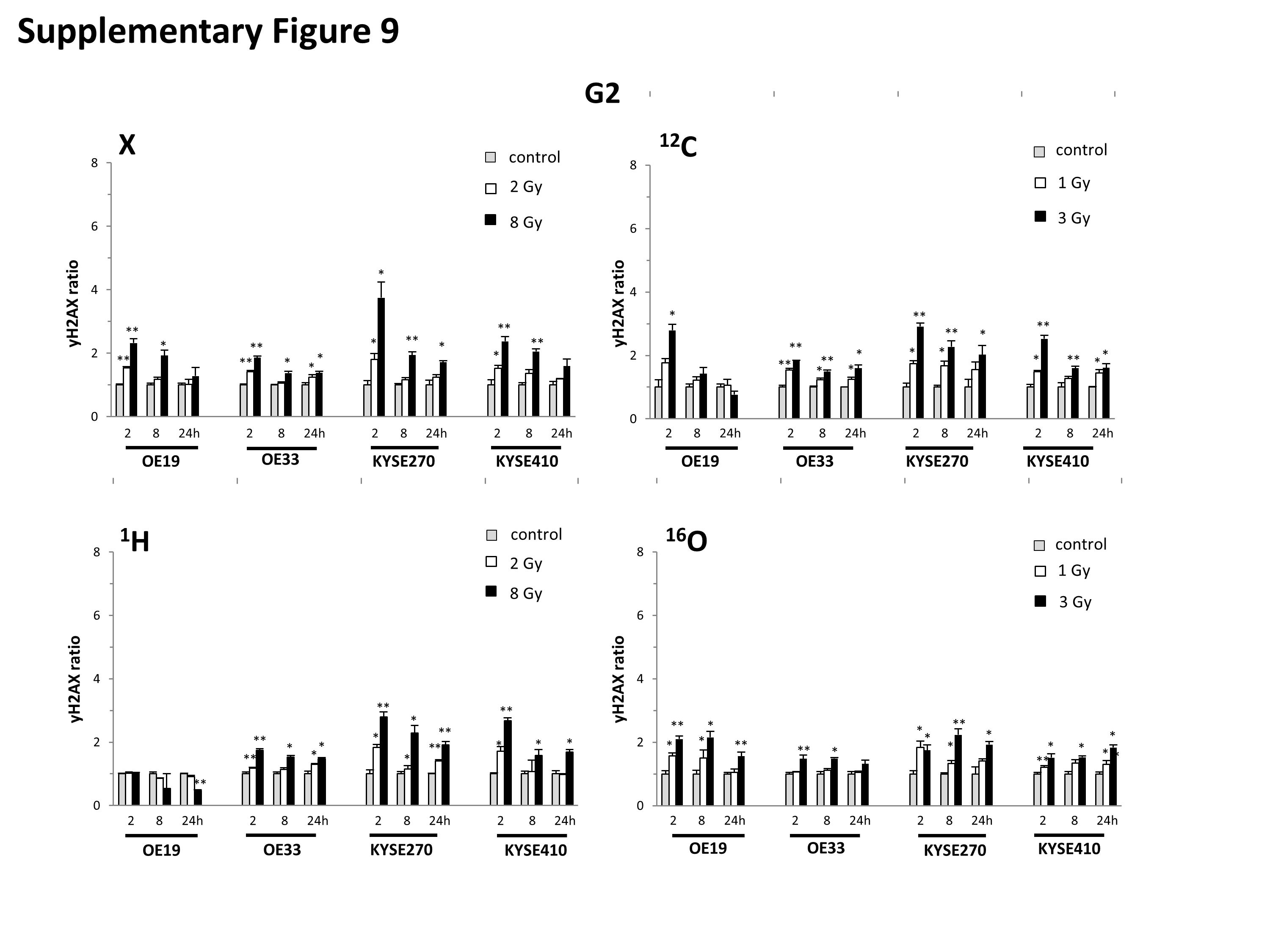

Supplement: Supplementary file 9 — Figure S9. Induction and repair of DNA double strand breaks in G2 phase cells after irradiation. Normalized γH2AX levels of G2 phase cells at 2, 8 and 24 h after irradiation with biologically isoeffective doses of photons (X), protons (1H) and heavy ions (12C, 16O) (mean and SD of n = 3 replicate samples). *p < 0.05, **p < 0.01 (two-sided Student’s t-test against unirradiated controls). (TIF 568 kb) [file 13014_2019_1326_MOESM9_ESM.tif]

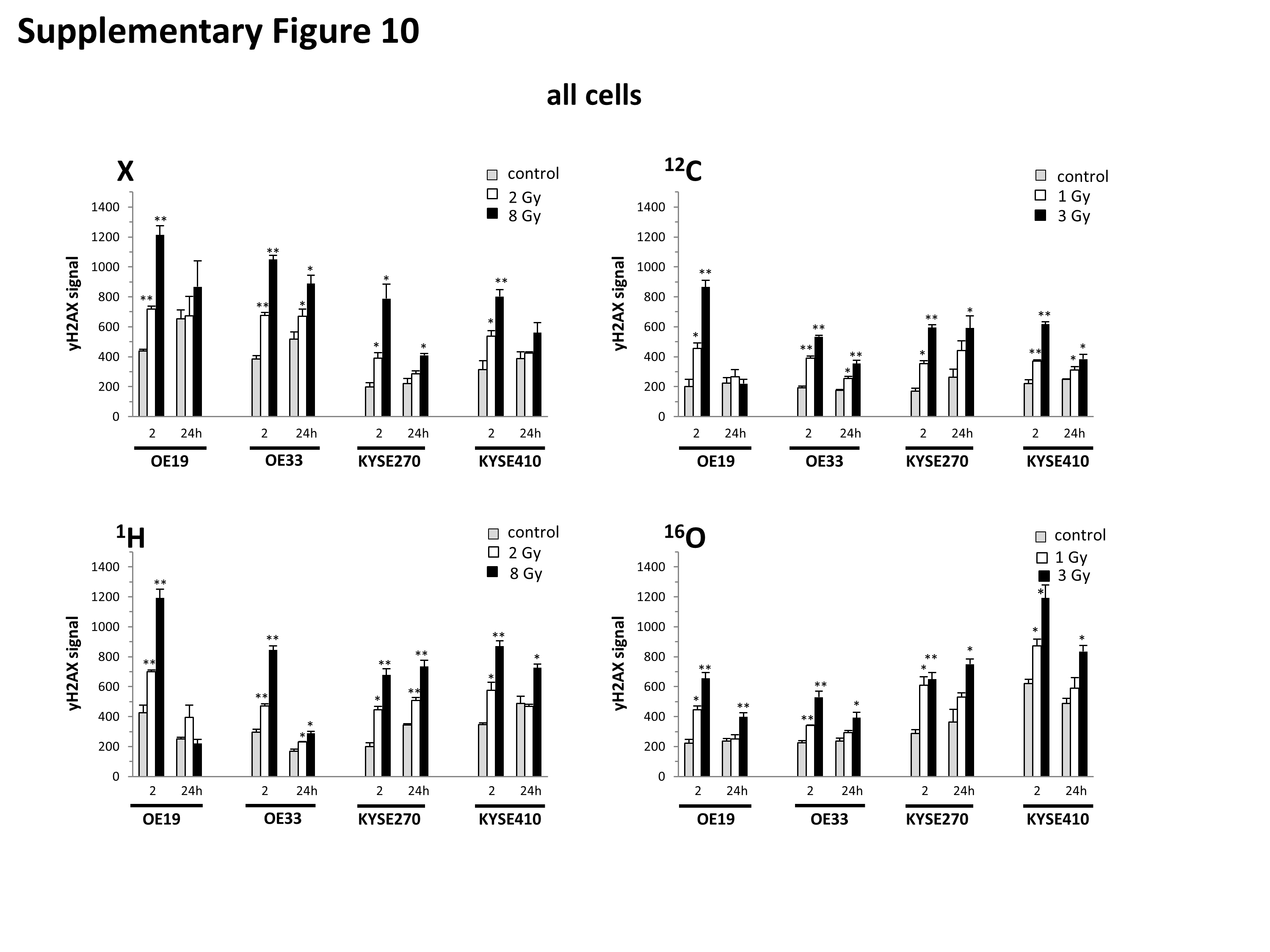

Supplement: Supplementary file 10 — Figure S10. Induction and repair of DNA double strand breaks in esophageal cancer cells after irradiation. γH2AX levels (not normalized) at 2 and 24 h after irradiation with biologically isoeffective doses of photons (X), protons (1H) and heavy ions (12C, 16O) (mean and SD of n = 3 replicate samples). Values were corrected for cell cycle-specific differences as detailed in Materials and Methods. *p < 0.05, **p < 0.01 (two-sided Student’s t-test against unirradiated controls). (TIF 602 kb) [file 13014_2019_1326_MOESM10_ESM.tif]
